# Influence of amyloid beta on impulse spiking of isolated hippocampal neurons

**DOI:** 10.3389/fncel.2023.1132092

**Published:** 2023-04-13

**Authors:** Volodymyr A. Yavorsky, Nataliia M. Rozumna, Elena A. Lukyanetz

**Affiliations:** Department of Biophysics of Ion Channels, Bogomoletz Institute of Physiology, National Academy of Sciences of Ukraine, Kyiv, Ukraine

**Keywords:** amyloid, rhythmic spiking, action potential, tonic generation, patterns of generation, perforated patch clamp, isolated hippocampal neurons

## Abstract

One of the signs of Alzheimer’s disease (AD) is the formation of β-amyloid plaques, which ultimately lead to the dysfunction of neurons with subsequent neurodegeneration. Although extensive researches have been conducted on the effects of different amyloid conformations such as oligomers and fibrils on neuronal function in isolated cells and circuits, the exact contribution of extracellular beta-amyloid on neurons remains incompletely comprehended. In our experiments, we studied the effect of β-amyloid peptide (Aβ1–42) on the action potential (APs) generation in isolated CA1 hippocampal neurons in perforated patch clamp conditions. Our findings demonstrate that Aβ1–42 affects the generation of APs differently in various hippocampal neurons, albeit with a shared effect of enhancing the firing response of the neurons within a minute of the start of Aβ1–42 application. In the first response type, there was a shift of 20–65% toward smaller values in the firing threshold of action potentials in response to inward current. Conversely, the firing threshold of action potentials was not affected in the second type of response to the application of Aβ1–42. In these neurons, Aβ1-42 caused a moderate increase in the frequency of spiking, up to 15%, with a relatively uniform increase in the frequency of action potentials generation regardless of the level of input current. Obtained data prove the absence of direct short-term negative effect of the Aβ1–42 on APs generation in neurons. Even with increasing the APs generation frequency and lowering the neurons’ activation threshold, neurons were functional. Obtained data can suggest that only the long-acting presence of the Aβ1–42 in the cell environment can cause neuronal dysfunction due to a prolonged increase of APs firing and predisposition to this process.

## 1. Introduction

Alzheimer’s disease (AD) is the most common form of progressive dementia with persistent, progressive memory and cognitive impairment and plays a leading role among late-onset diseases. Multiple causes of AD have been shown, but only 3–5% of AD cases are due to genetic factors (Foidl and Humpel, 2020; Zhang et al., 2020), while other cases have a sporadic onset. Health and environmental factors play a significant role in the development of AD, for example, the effect of hypertension on the development of AD (Carnevale et al., 2016), in which high blood pressure disrupts the brain barrier, type II diabetes, hyperinsulinemia, hypercholesterolemia, obesity; cerebrovascular damage in hypoxia and stroke, excessive activation of homocysteine, intoxication with heavy metals, including copper and iron ions (Singh et al., 2013; Becerril-Ortega et al., 2014). The treatment of AD is based on a various hypotheses, which pay attention to the mechanisms of disease development—deposition of amyloid plaques, hyperphosphorylation of tau proteins, damage of the cholinergic transmission, oxidative stress, impaired lipid metabolism, dysfunction of synapses and others.

The “amyloid” hypothesis associates the disease’s development with the accumulation of senile plaques, which mainly consist of amyloid-β (Aβ) aggregates (Querfurth and LaFerla, 2010). Plaques are formed in the extracellular space between neurons under overproduction of Aβ and amyloid precursor protein (APP) by neurons. Enzymatic cleavage of APP by BACE1 and γ-secretase produces the neurotoxic peptide Aβ, which contains 37–42 amino acids. Aβ is excreted outside the neurons and can oligomerize into chains. Aβ1–42 aggregated into oligomers is considered particularly toxic (Cline et al., 2018). While tau dysfunction and tangles have been identified as more robust correlates with cognitive impairment in AD than amyloid plaques, it is still vital to study the role of plaques in the pathogenesis of this disease. Amyloid plaques are a hallmark pathological feature of AD, and their presence has been associated with the onset and progression of the disease. Thus, studying the role of amyloid plaques is still essential for a comprehensive understanding of the disease pathogenesis and for developing effective treatments. In recent studies on the role of Aβ in AD discussed the hypothesis that Aβ accumulation leads to neuronal dysfunction and cognitive impairment. The authors of the review highlight evidence from both animal models and human studies suggesting that Aβ can disrupt neuronal signaling, cause inflammation, and impair synaptic plasticity, ultimately leading to neurodegeneration (Palop and Mucke, 2016). Moreover, the hippocampus (the brain structure responsible for learning and organizing memory) is the core structure that undergoes the first and most significant changes in AD development (Morris et al., 1982; Burgess et al., 2002; Kazim et al., 2017).

Currently, the effect of excessive Aβ production on neural function and network is a highly relevant topic. According to a study by Morimoto et al. (1998), Aβ injection experiments in rat hippocampus demonstrate significant amyloid accumulation around the hippocampus, accompanied by considerable neuronal damage in the CA1 and CA4 regions, as well as the dentate gyrus. Similarly, injection of Aβ1–42 into the rhesus macaque brain resulted in intercellular accumulation of Aβ plaques and neuron death, including cholinergic neurons (Li et al., 2010). Intracellular accumulation of Aβ is also possible in the pathogenesis of AD, and in the initial stages precedes the appearance of amyloid deposits outside the cells (Gyure et al., 2001; Bossers et al., 2010; Friedrich et al., 2010). Such accumulation can cause cognitive impairment even before the stage of amyloid plaques (Billings et al., 2005) and can cause hyperexcitation of neurons (Scala et al., 2015). APP and its fragments, including Aβ, were found on the endoplasmic reticulum membranes, Golgi apparatus, endosomal, lysosomal, and mitochondrial membranes (Kravenska et al., 2020). According to the amphipathic nature of Aβ, it is possible to assume its affinity for the membrane, and therefore the possibility of penetration or attachment to the surface of membranes with the “carpet” or “detergent” effect of damage (Bharadwaj et al., 2018).

The mechanism of Aβ action is still not fully understood, with the loss of membrane integrity being the most commonly considered mechanism. It was also supposed that the Aβ peptide can form a pore in the membrane, which can lead to cell death by affecting cell potential. According to this “pore” hypothesis, the Aβ channel formed by Aβ is permeable to calcium cations in anionic lipids and acid solutions (Hirakura et al., 1999). An excessive influx of calcium through these channels can overload the cell with calcium, resulting in apoptosis and eventual cell death. Aβ in the aggregate state can also cause a decrease in membrane fluidity and, consequently, have a detrimental effect on cell function. According to another hypothesis, Aβ can cause the oxidation of neuronal lipids and leads to the excessive production of free radicals (Butterfield et al., 2001).

Hypotheses about the effect of Aβ on the neuronal generation of action potentials (APs) deal with a wide range of mechanisms acting on different organizational levels of neural networks. On the level of channel structures, studies show that Aβ is a physiological modulator of various currents due to the effect on the expression or activity of channel proteins: A-type potassium current through Kv4 channels, HCN channels, sodium Nav1.1 currents (Plant et al., 2006; Ping et al., 2015). Aβ can block the α7 and α4β2 subunits of the nicotinic acetylcholine receptor (nAChR) in hippocampal cells and cause a presynaptic increase in calcium concentration (Dougherty et al., 2003). The effect of Aβ can also be mediated through the M-current of KCNQ channels (Kv7) (Hessler et al., 2015), corresponding to the data about cholinergic deficiency in the development of AD with memory impairment when blocking cholinergic mechanisms. Intracellular calcium levels go up in transgenic models of mice with AD; such calcium loading can provoke cell death (Querfurth and LaFerla, 2010).

According to another mechanism, chronic exposure to Aβ significantly reduces the branching and length of neurites due to massive damage to synapses and thus increases neuronal bodies’ input resistance and thus enhances the response to stimuli (Ghatak et al., 2019). Besides, various Aβ effects on different subcellular compartments (axon, dendrites, and mitochondria) were detected (Kravenska et al., 2016, 2020; Li et al., 2017). The indirect effect of Aβ on neurons can be developed by losing certain types of neurons, such as acetylcholinergic neurons, through inflammation, glial dysfunction, and deposition of Aβ inside blood vessels and their damage, known as cerebral amyloid angiopathy. Similarly, the specific effects of Aβ are associated with dopaminergic FS interneurons in the early stages of AD (Martorana and Koch, 2014).

The effect of Aβ is mainly classified as hyperexcitation of the neuronal network due to either increased excitability of neurons or changes in excitatory and inhibitory transmission between neurons. Damage of synaptic inhibition or excitation/inhibition balance leads to increased gamma-oscillatory activity, the formation of hypersynchronous activity in the neural network, and impaired plasticity of synaptic effects, and in turn, leads to cognitive abnormalities in AD (Mucke and Selkoe, 2012; Busche and Konnerth, 2016; Palop and Mucke, 2016). However, some studies suggest a decrease in interneuron firing under the influence of Aβ (Hazra et al., 2013) because inhibitory neurons have lost the ability to generate APs reliably, leading to hippocampal dysfunction for the APdE9 mouse model. The latter implies the amplification of sodium leakage currents for inhibitory neurons, with a decrease in the amplitudes of APs and depolarization of the resting membrane potential (Perez et al., 2016). Another study shows that Aβ reduces the impulse activity of fast-spiking interneurons (possibly parvalbumin-positive cells), and the effect differs for various types of interneurons (Villette et al., 2010). However, the relationship between interneuronal firing deficiency with cognitive dysfunction in AD has not yet been experimentally proven. It is suggested that synaptic inhibition may lead to a deficiency of neural network deactivation, promote epileptiform activity, and reduce gamma- and theta-oscillatory activity in patients with AD (Palop and Mucke, 2016).

All these hypotheses and various responses at different organizational hierarchical levels of the nerve system create difficulties in proving the direct effect of Aβ, as there is a whole system of changes in the functioning of neural networks, which cross-initiate other changes. Common Aβ disturbances in the brain are mainly investigated at the brain slices level. In contrast, the study of effects at the single-cell level is limited. Many relationships and correlations do not show the direct effect of Aβ on individual neuron, so there is a need for particular experiments on isolated neurons that can explain the mechanisms of Aβ effects.

The study of the impulse activity of neurons in current-clamp mode was carried out previously with using the extracellular application of Aβ (Jhamandas et al., 2011; Hector and Brouillette, 2020) or the intracellular actions of Aβ through the patch-pipette solution (Fernandez-Perez et al., 2021).

However, the experiments were carried out on cultured cells or brain slices, which had limited isolation from the influence of branched neurites or synapses. These factors have a significant effect on the membrane potential fluctuations of the soma, which could not be adequately compensated in the experiments. Consequently, the shifts in the membrane potential could influence the pulse generation and lead to indirect alterations in the firing frequency.

Several studies were focused on the impact of chronic exposure to amyloid-β on neuronal activity. Although they also did not isolate neurons, these studies emphasized the need for precise control of holding potential, fixing it at −65 or −80 mV (Tamagnini et al., 2015b; Wang et al., 2016; George et al., 2021). However, the testing of neurons for intrinsic excitability were performed using a pipette solution that washed out some intercellular components of the soma, leading to possible alterations in the cell cytosol and resulting in a rapid deterioration of spiking properties.

In summary, the effect of Aβ on neurons is an enhancement in spike firing activity, which is known as hyperexcitability. Hyperexcitability is characterized by an increase in the number of repetitive APs in response to a depolarizing input current stimulus (Jhamandas et al., 2011). It was also regarded as increased excitability and loss of accommodation properties (Jhamandas et al., 2001). The firing threshold of neurons decreased in response to oligomeric Aβ treatment, resulting in the initiation of spikes at lower current stimuli, indicating a reduction in rheobase for firing (Fernandez-Perez et al., 2021). The voltage-dependent decrease in membrane conductance can explain the latter and the acute response of neurons to Aβ1–39, Aβ1–28, and Aβ1–40 by blockage of K+ channels (Good et al., 1996), as well as by an increase in membrane input resistance R_*in*_ (Tamagnini et al., 2015a). However, the authors imply the long-term compensatory changes due to the increased excitability that brings back resting potential and R_*in*_ to physiological levels. Incubation of cultured neurons with oligomeric Aβ 42 increases an action potential firing rate (George et al., 2021). Hyperexcitability was also attributed to the hyperpolarization of the action potential threshold after Aβ 500 nM treatment (Tamagnini et al., 2015b), but without any alterations in sub-threshold intrinsic properties of neurons, including membrane potential and input resistance. A lower threshold and a more prominent action potential burst were recorded on primary cultured pyramidal neurons after Aβ1–42 chronic exposure (Wang et al., 2016).

Several studies have noted the complex effects of Aβ on neuronal activity. While some suggest that Aβ initially increases neural excitability, compensatory inhibitory mechanisms may lead to hypoactivity (Hector and Brouillette, 2020). However, this is likely due to the effect of Aβ on synaptic mechanisms rather than on the neuron’s soma. Another study (Spoleti et al., 2022) has shown that Aβ can impair cell excitability in CA1 hippocampal neurons in Tg2576 AD mice, that can be attributed to synaptic dysfunction in the hippocampus in this model.

There are two proposed mechanisms that suggest Aβ may increase the generation of action potentials in nerve cells. Aβ causes changes in the characteristics of APs discharges on the soma of neurons, and on the other, Aβ specifically impairs synaptic transmission (Ren et al., 2018).

Although the primary suspect for the leading role is synaptic disorder, the mechanisms must be compared and quantified since there is insufficient data on the influence of Aβ on the activity of individual neurons. Therefore, our study aimed to determine the direct effect of Aβ1–42 on APs generation in isolated neurons of the rat hippocampus by using the perforated-patch clamp experimental design and outline the effects of Aβ1–42 on neurons without synaptic influences by using the analysis based on the construction of the activation APs functions based on the data obtained on isolated neurons.

## 2. Materials and methods

### 2.1. Isolation of neurons of the CA1 zone of the hippocampus

All animal treatment and care procedures were conducted following the National Academy of Sciences of Ukraine and Bogomoletz Institute of Physiology guidelines for the care and use of laboratory animals. The method of obtaining isolated neurons of the rat hippocampus generally corresponded to the method described in our previous works (Iavors’kyi and Luk”ianets’, 2013; Yavorsky and Lukyanetz, 2015). Animals (30 rats of 14-day-old) were decapitated after anesthesia with ether; the brain was quickly removed and transferred to a cold (4°C) solution A. Slices of the hippocampus 0.4–0.5 mm thick were cut with a blade and then kept for 60 min in solution B at room temperature (21–25°C), placed them on a nylon mesh in the chamber; aeration of the medium was provided by carbogen. Enzymatic treatment in solution-B with 0.1% pronase (type 23) and 0.1% trypsin (”Sigma,” USA) lasted 20–35 min without changing the ambient temperature. Dispersion of hippocampal slices allowed us to obtain isolated neurons of the required zone, which preserved the small parts of the apical and basal dendrites and had a soma with a diameter of 15–20 μm.

Solution-A contained (in mM): NaCl 120, KCl 5, HEPES 10, MgCl_2_ 1, CaCl_2_ 2, glucose 25. Solution-B (in mM): NaCl 125, KCl 5, NaH_2_PO_4_ 1.25, NaHCO_3_ 25, MgCl_2_ 1, CaCl_2_ 2, glucose 10. The pipette solution (in mM): C_6_H_5_O_7_K_3_ (tripotassium salt of citric acid) 60, KCl 20, HEPES 10, MgCl_2_ 5; amphotericin-B, pre-dissolved in DMSO, was added to the pipette solution at a final concentration of 1 mg/ml. Caffeine (a tool to test intracellular calcium signaling), a blocker of sodium conductance quinidine, selective M1 and M2 agonists McN-A-343 and muscarinic cholinergic agonist oxotremorine, and other substances were obtained from Sigma-Aldrich, USA. Quinidine was tested as a blocker of sodium conductance, as well as two specific blockers of potassium conductance, to determine how effects on membrane conductance alter APs generation. The effects of caffeine were seen through intracellular mechanisms, through the endoplasmic depot, and through calcium mechanisms.

### 2.2. Electrophysiological leads and protocols

Transmembrane currents and potentials were measured using standard patch-clamp techniques under conditions of perforation of the membrane region under the action of amphotericin-B (Korn et al., 1991; Iavors’kyi and Luk”ianets’, 2013; Yavorsky and Lukyanetz, 2015). Briefly, patch pipettes were filled with the pipette solution containing 5mg/ml amphotericin-B, previously dissolved in DMSO. After 5÷15 min of tight contact (more than 3GΩ) with the cell membrane, the serial resistance decreased to 4÷10 MΩ due to membrane perforation by the antibiotic. The cells were considered “healthy” and were brought into the experiment if their stable resting potential was more negative than −50 mV and the action potential showed an overshoot. Then we compensated the Ve of the amplifier to set the neuron’s resting membrane potential to −80 mV. Cell activity usually became sufficiently stable about 20 min after starting the clamping, with a duration of 20–30 min for recording. The series of 4–5 ramp protocols have applied a minimum twice for the control solution, tested solution and rewashed by control solution, with 2–3 min resting interval for every cell between series. All recordings were performed at room temperature.

After the seal on the patch pipette, the isolated neurons were lifted from the surface of the experimental chamber to prevent them from touching other cells or resting at the bottom of the chamber. The neurons remained submerged in a solution with a constant laminar flow rate. Aβ1–42 was administered to the isolated neuron using an application pipette with an inner tip diameter of approximately 0.5 mm, about 4–5 mm away from the cell. Mainly, only the data from the initial application of Aβ1–42 was included in the analysis due to incomplete recovery during the washing procedure. The control and testing solutions and the isolated neuron were replaced to prepare for subsequent testing procedures. Due to our stringent selection criteria for AP firing properties, only one neuron was successfully recorded per experimental day, with numerous cells being rejected. A total of 10 records were accepted for further analysis. Changing the extracellular solution’s composition was performed by switching the flow with the preservation of a constant flow rate into the chamber while pumping the existing solution with a peristaltic pump on the other side. The duration of the Aβ1–42 application did not exceed 15 min. Introducing low DMSO concentrations (0.1–0.5%) into the extracellular solution does not alter the generation parameters because DMSO was already present in the pipette solution due to the perforated patch method. The PC-ONE amplifier (Dagan Corp., USA) and the Digidata 1200 board (Axon Instruments) were controlled by the software “WinWCP” (University of Strathclyde, UK); patch pipettes were produced on a P97 puller (Sutter Instruments, USA).

Lyophilized Aβ1–42-amyloid rat (SCP0038 Sigma, USA) was diluted in dimethyl sulfoxide to get 1 mM stock solution and stored at a temperature of –20°C. Test solutions with Aβ1–42 were prepared on the day of use by adding Aβ stock into control solution-A to get concentrations from 200 nM to 10 μM and then were stored at room temperature for 1–4 h. The final concentration of dimethyl sulfoxide did not exceed 0.1%. The amyloid fibrils used in the experiments were predominant in aggregated form of Aβ1–42. Amyloid aggregation was verified by measuring thioflavin T fluorescence using a Hitachi F-4000 fluorescence spectrometer. The presence and formation of a fibrillar peptide were indicated by an increase in the fluorescence of thioflavin T (excitation at 450 nm, recording the intensity of fluorescence at 480 nm). Also, we detected the acute shift of extinction coefficient at 415 nm for solution of Aβ1–42 20 μM with thioflavin T 10 μM during the period 1–1.5 h of incubation at 37°C (absorption spectrum with NanoDrop 1000, Proteine&Label protocol, drop volume 2 μl), which may indicate Aβ fibril formation.

Reproducibility of APs generation was achieved by selecting cells with stable characteristics, unique tuning of stimulation protocols, and constant monitoring of the resting potential in neurons with stabilization of the latter at the same level. Toward the selection of protocols that characterize the regular pulse generation and control the pulsing degradation, we proposed serial testing of cells by rectangular and “ramp”-like stimuli (Iavors’kyi and Luk”ianets’, 2013; Yavorsky and Lukyanetz, 2015). Test stimulation with a rectangular waveform consisted of a series of 12 rectangular stimuli at a holding resting potential of membrane −80 mV, with a duration of a single stimulus of 1 s. Neurons responded to stimulation by generating APs, which made it possible to estimate neuronal accommodation properties. Records of membrane potential from the 3rd to the 12th testing pulse were used for the analysis. The APs firing in the first and second pulse usually differed slightly from subsequent firing due to the accommodation effect. We apply ten-second resting intervals between stimuli in series. If necessary, manual adjustment of the resting potential was performed to achieve stable conditions during the long-lasting testing of neurons in the periods between stimuli. Test stimuli of triangular shape (ramp testing) lasting 5 s were repeated in series 3–5 times, with 15–20 s between stimuli.

### 2.3. Construction of activation functions of APs generation of neurons

The functional test for pulse generation changes in a neuron is a sensitive tool for detecting the effects of Aβ. The effect of Aβ1–42 exposure was defined as changes in the APs generation of neurons in terms of voltage recordings: the shift of the activity threshold, frequency of induced generation, number of repeated APs upon stimulation, AP half-width (Ghatak et al., 2019). However, insufficient control of the conditions under which the cell generates APs causes shortcomings in most studies’ testing schemes. Thus, determining activity by the delay time of AP generation in response to the input stimulus may be uninformative without control of the initial resting potential. The shift of this potential can cause significant errors in determining the parameters of APs activity. Besides, there is absolute randomness of generation in repeated recordings. More informative is a series of tests with the same stimulus, where the input current slowly increases over the transmembrane potential level. APs time samples can be calculated from the record and converted into inter-peak intervals or instantaneous APs frequency. Furthermore, it is worth noting that membrane resistance of the neurons was not directly measured during patch-clamp recording using a specific built-in protocol. Instead, it was calculated post-hoc from recordings of ramp protocols obtained before and after the application of Aβ1–42. To calculate the membrane resistance (Rm), we utilized a linear region of the recorded membrane potential (Vm) below the impulse spiking, while discarding the area near the spiking threshold due to the non-linear rise of Vm. We fitted the linear region to a linear function, and converted the slope parameter into resistance data, assuming that slow changes in the incoming current reflect mostly passive properties of the membrane between −80 and −65 mV. However, it’s important to note that the potential slope parameter is a variable, particularly when passing through the generation threshold, and may be influenced by factors such as the quality of the recording or uncontrolled leakage currents.

Also, the input resistance is not a characteristic value for a particular group of neurons but rather an individual characteristic of the neuron. It is important to note that the whole-cell configuration used for registering neuronal activity may not be optimal due to the effect of “washing out” the internal composition of the neuron in a pipette solution. This is because the whole-cell configuration involves rupturing the cell membrane, which may alter the intracellular environment and introduce confounding factors. Additionally, the pipette solution used for whole-cell recordings may differ in composition from the internal environment of the neuron, further complicating the interpretation of results. The latter causes a constant change in the pulse generation’s characteristics with a “rundown” activity in a few minutes. The latter leads to the absence of a period of constancy of the excitation characteristics in that it is possible to test the Aβ. Therefore, we used a configuration of perforated patch-clamp. In this case, the washout effect is much smaller, and there is a period in which pulse generation has almost constant characteristics. Thus, an informative method of analyzing the neuron’s impulse activity is to build the APs activation functions of neurons in the protocol with a slow increase in input current (”ramp” protocol) under conditions of quality control and determination of cell generation status (Yavorsky and Lukyanetz, 2015). We determined the peak times of AP and calculated the instantaneous APs frequencies as inverse values of the APs intervals. The APs function was constructed on the graphs as a sequence of frequency values (in the ordinate) depending on the average input current at the inter-peak interval (abscissa axis). Such average current values were calculated from the input current’s magnitude, which corresponds to the middle of each APs interval. Elementary linear transformations of APs functions were used to determine the frequency and input current axis shift factors. The APs function was constructed on the graphs as a sequence of frequency values (in the ordinate) depending on the average input current at the APs interval (abscissa axis). Such average current values were calculated from the input current’s magnitude, corresponding to the middle of each peak interval. The multiplication factor of the APs function was set as the value that most successfully reproduced the shape of the control sequence from the Aβ-sequence after multiplying the input current values.

We evaluated the neuronal ability to generate APs in the “ramp” protocol and selected only those cells that could generate the APs in a wide range of input current intensities, usually under stimulus from 10 to 100 pA. Also, an analysis of the generation dispersion was performed. Any cell failing to meet the selection criteria was excluded. These criteria included having a well-defined body shape with no indications of swelling, a resting potential that is consistently more negative than −50 mV, action potential demonstrating an overshoot, multiple AP firing that is sustained in response to inward current stimuli, and a neuron that exhibits rhythmic firing with a relatively low coefficient of variation (CV) of interspike intervals less than 30%.

## 3. Results

**FIGURE 1:**
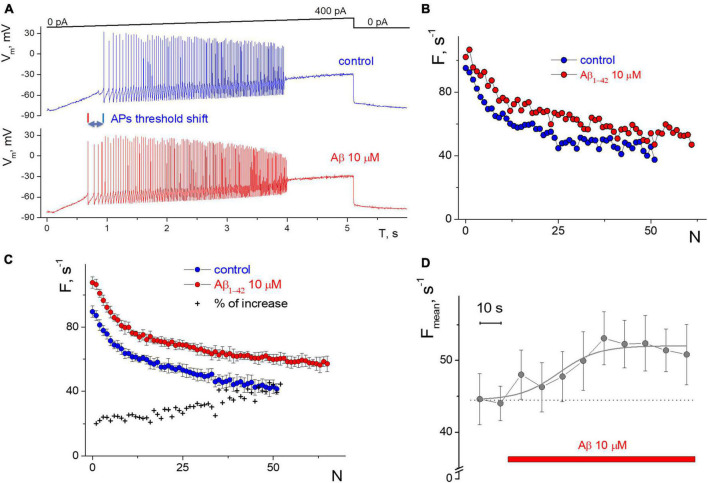
Influence of 10 μM Aβ1–42 on the induced impulse generation of a neuron compared to control. **(A)** Records of the neuronal membrane potential in control and under the Aβ1–42 action in the “ramp” protocol with a slow increase in the input current from 0 to 400 pA. **(B)** Firing frequency sequences with accommodation effect in control and 60 s after the Aβ1–42 application in the protocol with a rectangular current impulse with an amplitude of 250 pA and 1 s duration. On the abscissa axis—the ordinal number of the AP, on the *y*-axis—the instantaneous frequency of APs generation (the inverse of the interval between successive AP). **(C)** Neuron’s firing frequency sequences with accommodation effect in rectangular protocol with an amplitude of 250 pA and 1 s duration, averaged over a series of 10 test impulses. Comparison of averaged control sequence and those after 6 min of the Aβ1–42 treatment, on the abscissa axis—the ordinal number of the AP, on the *y*-axis—the instantaneous frequency of APs. **(D)** The average frequency of pulse generation with a testing period of 10 s (mean ± SD over calculation interval from 0.7 to 1 s of each test impulse).

The neuronal firing activity was studied based on the frequencies of firing rate, the ability to generate APs at low and high amplitudes of the testing current, and the rhythmicity of firing, as was described previously (Yavorskii et al., 2006; Yavorsky and Lukyanetz, 2009, 2015; Iavors’kyi and Luk”ianets’, 2013). APs firing of isolated hippocampal CA1 neurons were obtained in the “ramp” protocol, the records from the fast-spiking neuron shown on Figure 1A. It is shown that Aβ1–42 enhances the neuronal capacity to generate action potentials in response to a broader range of stimuli. The latter means that the minimum threshold required for firing activation has decreased, and the maximum threshold for firing inhibition has increased compared to the standard firing. From the “ramp” records, we calculate the membrane resistance below the APs generation threshold by assessing the growth of membrane potential relative to the increase of input current. Neurons have Rm = 1.5 ± 0.6 GΩ in control, and Àβ1–42 elevates Rm by 16 ± 13% (mean ± SD, *n* = 10, *p* = 0.0015 for paired sample t-test), with minimal 4% and maximal 50% of the increase. The noticeable increase in Rm occurred in the first 2–3 min of Àβ1–42 application by 13 ± 15% (*n* = 8, *p* = 0.02). Washing out from Àβ1–42 reduced the Rm on average by 2 ± 19% nonsignificantly (*p* = 0.3), from a decrease of 35% to a further increase of 38%.

First, we consider the values of the instantaneous frequency of APs according to the input current stimulus intensity but previously check the signs of rhythmic firing. The selective approach was used because most tested neurons (>60%) showed a sporadic generation of APs irrespective of stimulation level. Their values of the instantaneous firing frequency formed a “cloud” of points with a significant variance on the graph, making it impossible to construct the dependence of the activation function and its analysis. For the registered “sporadic” neurons, we determined a significant variability within a series of test stimulations for the APs generation threshold (from 30 to 50 pA with repeated stimuli); also, the upper inhibition of APs generation occurred in a wide range of stimulus intensity, from 140 to 170 pA. A thorough analysis of the impulse firing of such neurons is impossible due to the low reproducibility of generation, which causes the unclear influence of external factors. It cannot be ruled out that sporadic firing activity may result from the cell’s apoptotic state, damage to the cell membrane after isolation from hippocampal sections, or excess calcium entry into the cell by activating pathological processes. Therefore, we excluded such neurons from the analysis. However, according to our observations, applying Aβ1–42 affects tested “sporadic” neurons with a decrease in the average threshold of APs generation by 5–10 pA.

The study of the action of exogenous Aβ1–42 on the impulse activity of isolated neurons CA1 of the hippocampus was performed at peptide concentrations from 200 nM to 10 μM. Such concentrations are significantly higher than the mean level of Aβ in amylodopathies at 0.5 nM (Waters, 2010). However, due to the aggregation of native amyloid into fibrils and plaques, the concentration of soluble or oligomeric Aβ1–42 can increase significantly near plaques and have a more intense effect on neurons (Tamagnini et al., 2015b). In order to demonstrate the saturation of Aβ1–42 on neuronal impulse activity, concentrations deemed sufficient were used. The specific fractions of aggregated Aβ or Aβ oligomers, which can differentially affect the firing of action potentials, are insignificant as all fractions were present in abundant quantities.

Also, the selected “rhythmic” neurons in the step protocol develop a moderate or low dispersion in the APs sequence, and this rhythmicity was checked under control conditions for 3–5 min before testing. We succeeded in recording APs activity with a narrow spectrum from nine neurons; the coefficient of variation was less than 30%, even with low-intensity stimulation. The low variance also allowed us to clearly distinguish the APs sequences in control and under the influence of Aβ1–42 (Figures 1B, C). Neuronal resting potential undergoes only a minor hyperpolarization up to 5 mV when the replacement occurs from control to Aβ1–42 solution, as well as slow spontaneous fluctuations of the membrane potential of neurons by 2–3 mV in amplitude. We manually compensated for this potential shift to −80 mV level to achieve constant conditions for testing and avoid the influence of the holding potential level on the APs frequency.

The direct effect of 10 μM Aβ1–42 was obtained in a series of stimuli by a rectangular current pulse with an amplitude of 250 pA, 1 s duration, and an interstimulus interval of 10 s. Aβ1–42 administration for 60 s caused increasing in firing frequencies by 20–30% (Figure 1B), with a minimal change in firing rate at the beginning of the stimulus. The accommodation effect of firing causes a gradual decrease in firing frequency during prolonged excitation. Accommodation shape is preserved under the influence of Aβ1–42, along with the characteristic temporal reduction in the APs firing frequencies. A further examination of the neuron firing’s properties showed only slight changes in generation frequencies that occurred from the second to 6th minute of Aβ1–42 application (Figure 1C), i.e., a prime shift in APs rate arises during the first minute of Aβ1–42 application. For the represented high-rhythmic neuron, the increase in frequency at the beginning of the stimulus was 20% and rose to the end up to 40%. The latter means that Aβ1–42 may influence a bit more significantly in case of long-lasting firing when the neuron experience accommodation. The count of APs in spike trains increased at Aβ1–42 relative to control, which indicates a diverse improvement in AP firing under the influence of Aβ. Also, we assessed the effect of Aβ1–42 on stationary firing and considered the average of APs firing frequency at the end of the rectangular stimulus, taking data from 0.7 to 1 s. Figure 1D shows the temporal influence of Aβ1–42 that causes a rise in the stationary frequency of APs firing values after 60 s. The influence effect was +20.5% compared to the control when manually controlling the resting potential toward −80 mV and reflecting the central part of the overall effect of Aβ1–42 for 10 min after application. Aβ1–42 elevates or maintains the APs rate for all neurons tested, even under significant inward current stimulation conditions.

**FIGURE 2:**
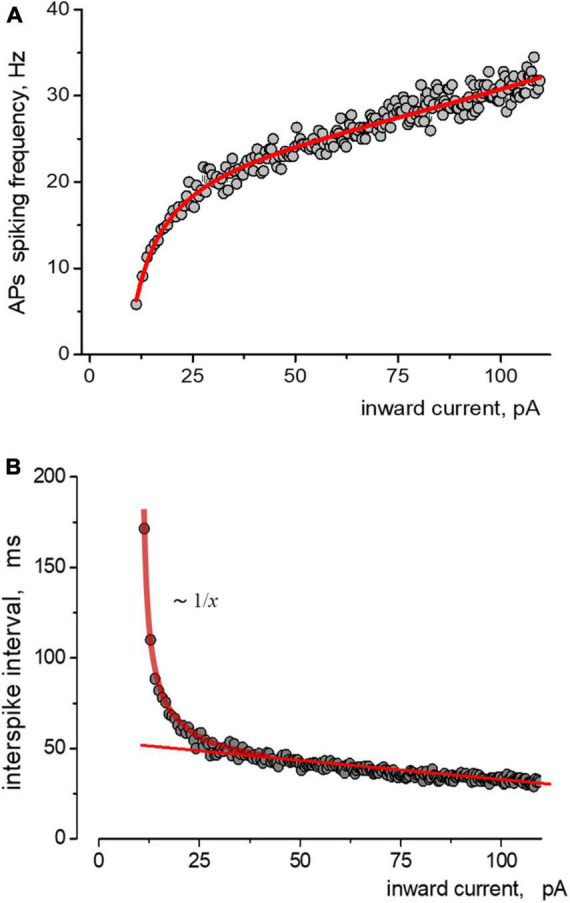
Approximations of the data obtained from induced impulse generation of a neuron. **(A)** Fitting the dependence of the frequency of spiking on stimulating inward current, where experimental data approximated by the Nelder function $y\,\left(x\right)=\frac{x+a}{b_{0}+b_{1}\,\left(x+a\right)+b_{2}\,{\left(x+a\right)}^{2}}$ were *a* = −9,1=0,6;*b*0=0,26=0,03;*b*1=0,040 + 0,001;*b*2 = −1,1*e* − 4 + 1*e* − 5. Points and solid lines present the experimental data and their approximations, respectively. **(B)** The same data but in form of dependence of the values of interspike interval on the amplitude of stimulating inward current is presented. Experimental data are shown as points, sequence is approximated by sum of reversal function *y(x) ∼* 1/*x* and a linear function.

The relationship between instantaneous APs firing frequency versus input stimulating current (current injection) we defined (considered) as the APs activation function of the neuron. Figure 2A depicts the relationship between the spiking frequency and the injection current, with experimental data points accurately represented by the Nelder equation. If we consider the spike intervals (the peak-to-peak) instead of the instantaneous frequency of AP generation, the Nelder equation reflects the sum of inverse proportional and linear functions, Figure 2B. The first function reflects the threshold component of APs firing with the threshold parameter a, while the linear function shows a proportional gain of APs generation at high input current intensity. The Nelder equation gives a good approximation for all of the APs trains in ramp protocols and thus the possibility to quantitatively estimate the effects of Aβ1–42 on the spiking activity of neurons.

**FIGURE 3:**
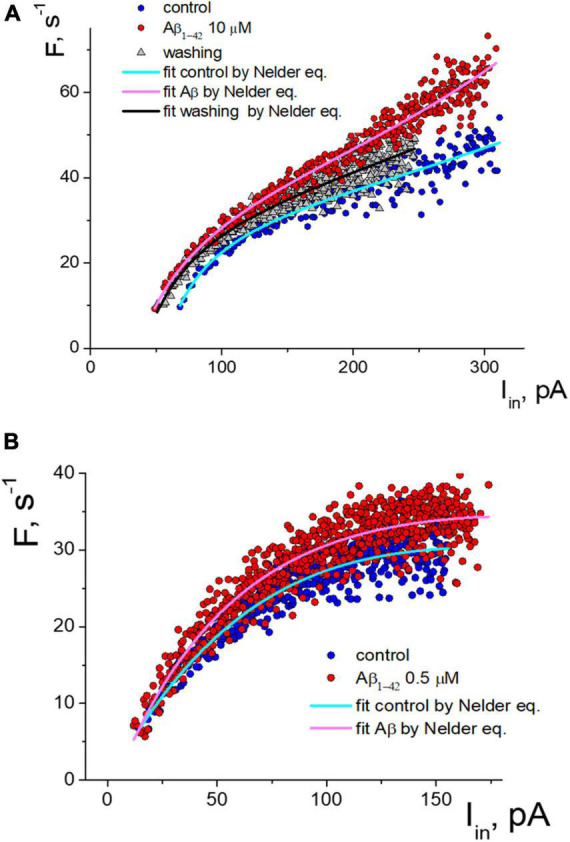
Two types of the response of pulsed generation of neurons to the application of Aβ1–42. **(A)** Change of an isolated neuron’s activity function with a decrease in the generation threshold under control and Aβ1–42 10 μM, and washing with a control solution which cause partial restoration of the activation function. **(B)** Aβ1–42 increase the frequency of APs generation of neurons without threshold shift. The approximation of instantaneous frequencies under control conditions and the action of Aβ1–42 is presented based on the Nelder function.

Under control conditions, the neuron began APs generation at a threshold current from 70 pA to more than 300 pA, with a corresponding increase in frequency from 10 s-1 to 45 s-1. Deviations of values from the primary trend of the sequence tend to be much smaller at the beginning of the stimulus (less than 5%). In comparison, the variance of values increases with intense stimulation (up to 20%). The application of 10 μM Aβ1–42 shifted the generation sequence, as seen in the scattering diagram (Figure 3A). Visually, the shift of values was observed upwards, and to the left side of the graph, so we analyzed two hypotheses about (1) an increase in firing frequencies and (2) a linear shift of sequence by input current corresponding to growth in effective depolarization. Using the Nelder equation, we estimated that a parameter (which reflects the threshold) was −56.5 pA in control, −36.0 pA at the influence of Aβ1–42, and −40.3 pA after washout. Thus, Aβ1–42 shifted the activation threshold in 20.5 pA toward the smaller values.

Detailed analysis showed that tested neurons are characterized by two types of responses to applying Aβ1–42. The shift of the generation threshold current towards smaller values was clearly expressed for six neurons (mean threshold 26 ± 22 pA, maximal frequency 29 ± 11 s-1, shift -41 ± 17%). AP generation under the influence of Aβ1-42 occurred at a lower stimulation intensity by the input current. At low amplitude of input current, the discrepancy in firing frequencies began much higher. For a neuron shown in Figure 3A, we observed the most significant frequency gain within the input current range from 70 to 150 pA. The effect was relatively minor for high-intensity stimulation due to the increase in the average firing frequencies. Washout from Aβ1–42 somewhat reduced the generation frequencies’ value, thus partially restoring the sequence toward the control sequence data. However, washout from Aβ1–42 significantly reduced the neuron’s ability to fire AP under intense stimulation, with a decrease in the suppression current threshold of AP firing by more than 20%.

In our experiments, four tested neurons had a different response to applying Aβ1–42, which was not accompanied by a shift in the firing threshold (mean threshold 15.5 ± 2.5 pA, maximal frequency 27 ± 7 s-1). An example of the activation data of such a neuron is shown in Figure 3B. For this group of neurons, Aβ1–42 caused a slight increase in the frequency of pulsed generation from 5 to 15%, with approximately the same increase in the firing frequency regardless of input current level. Under the high-intensity stimulus, a significant dispersion of the AP frequency values led to an overlap of the control sequence and the Aβ-sequence.

We define that Rm was changed similarly under Aβ1–42 for two groups of isolated neurons. In the group of neurons with firing threshold shift, Rm = 1.4 ± 0.7 GΩ in control and Aβ1–42 elevates Rm by 21 ± 15% (mean ± SD, *n* = 6, *p* = 0.01 for paired sample *t*-test), with minimal 7% and maximal 50% of the increase. Washing out from Aβ1–42 reduced the Rm on average by 8 ± 15%, from a decrease of 35% to a further increase of 6% (*p* = 0.16, insignificant). In the group of neurons with no firing threshold shift—Rm = 1.7 ± 0.5 GΩ and Aβ1–42 elevates Rm by 9 ± 6% (*n* = 4, *p* = 0.04 for paired -test), with minimal 4% and maximal 15% of the increase. Washing out from Aβ1–42 leads to multidirectional Rm changes with average nonsignificant growth by 9 ± 25%, from a decrease of 5% to an increase of 38%.

**FIGURE 4:**
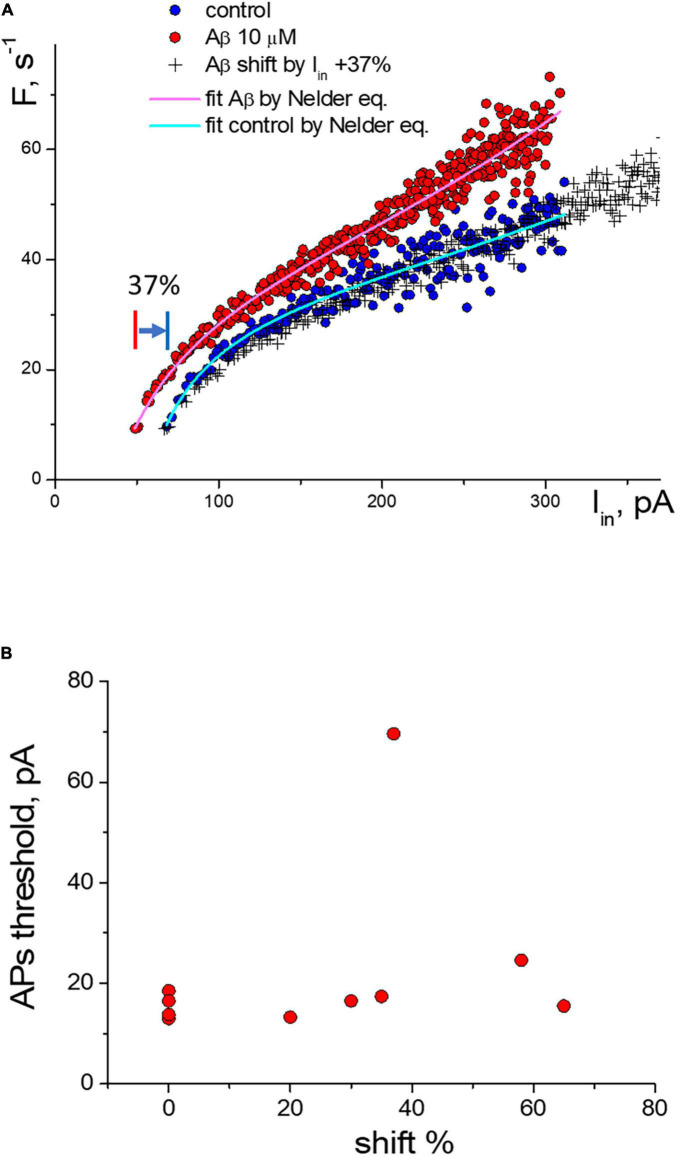
Change of the activation function of neurons along the input current’s axis under the influence of Aβ1–42. **(A)** Change of the activation function at 10 μM Aβ1–42 in comparison with the control activation function: (°)—control and Aβ1–42 sequences, (+)—transformed sequence obtained by stretching the input current values by 37% (multiplication factor *k* = 1.37). **(B)** Statistic scattering diagram of the generation threshold levels in control (pA) vs. multiplication (stretching) coefficients in % due to Aβ1–42 action for 10 neurons.

For the experimental neurons from the group with the threshold shift response, we found a clear overlay of frequency sequences in control and at Aβ1–42 when transforming Aβ-sequences by multiplying the input current values on the same coefficient (Figure 4A). We can see in the graph that both the control sequence and the Aβ-sequence nearly coincide when multiplying the input current of the Aβ-sequence (x values) by a factor of 1.37 (i.e., increase by 37%). Multiplying factors were roughly calculated for neurons as the firing threshold ratio in control and Aβ1–42 sequences. The multiplied Aβ1–42 sequence visually fully overlaps the control sequence at any input current. Accordingly, the calculated shift factor may resemble the effect of Aβ1–42 on the APs activation function of neurons, and actually, Aβ1–42 causes an uprise of the effective input current without form violation of the APs activation function. The multiplication factors diagram shows the values range from 20 to 65%, with a mean value of 41% (Figure 4B). We did not find a significant correlation between the values of the multiplying factor and the firing threshold current (Pearson *r* = 0.27), the maximal firing frequency (*r* = −0.03), the threshold current of firing suppression (*r* = −0.1), or the concentration values of Aβ1–42, which we used in the range from 200 nM to 10 μM. Presumably, the maximal effect of amyloid may vary between neurons depending on their type or condition or specific sensitivity to Aβ with shifting action.

**FIGURE 5:**
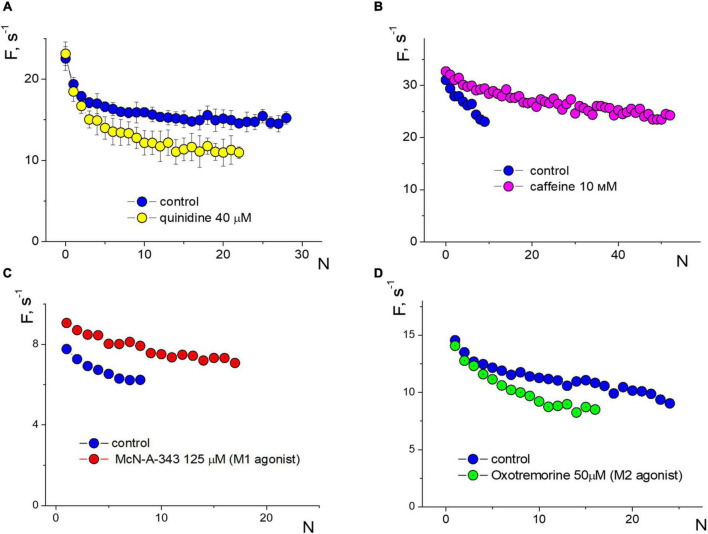
Changes in induced firing generation with accommodation effect of a neuron compared to control. Rectangular protocol applied with 1s duration. **(A)** Firing frequency sequences in control and under quinidine 40 μM, testing with an input current of 20 pA. **(B)** Firing frequency sequences in control and under caffeine 10 mM, testing with an input current of 40 pA. **(C)** Firing frequency sequences in control and under McN-A-343 125 μM, testing with an input current of 20 pA. **(D)** Firing frequency sequences in control and under oxotremorine 50 μM, testing with an input current of 20 pA.

We compared the effect of amyloid against several other substances on APs firing in isolated neurons (Figure 5). When stimulated with an input current of 20 pA, the voltage-sensitive sodium channel blocker quinidine 40 μM reduced the frequency of APs down to 25% during prolonged spiking but not at the beginning of the stimulation (averaged data over a series of 10 test stimuli, Figure 5A). Caffeine 10 mM, on the contrary, significantly reduced the effect of accommodation without changing the initial spiking frequency and increased the number of repeated APs upon stimulation of 40 pA (Figure 5B). Previously, we saw the remarkable effect of the epileptogenic substance pilocarpine on the firing of isolated neurons (Yavorskii et al., 2006), so we tested the effect of M1 and M2 muscarinic receptor agonists on APs function (Figures 5C, D, respectively). Under a stimulus of 20 pA, agonists show different directions of changes in APs frequencies and the slope of the APs sequence due to the accommodation effect. The M1 agonist McN-A-343 125 μM uniformly increased the APs frequency to 18–28%, while the M2 agonist oxotremorine 50 μM decreased the frequency of APs in a use-dependent manner without changing the initial APs frequency.

## 4. Discussion

Based on our data, it appears that Aβ1–42 has a dual effect on the impulse firing of neurons. Despite the different characteristics of these effects, they both lead to an enhancement of the firing response of neurons. This enhancement is particularly noticeable during the first minute after the application of Aβ, with a slight increase observed within 5–6 min. In conditions of perforated-patch mode, this proves the absence of direct damaging effects of significant concentrations of amyloid on neuronal functionality due to possible mechanisms of channel formation, reduced membrane fluidity, or loss of its integrity.

Moreover, Aβ1–42 improves the firing capacity of neurons, and we found no restrictions of this effect for cells with a greater or lesser maximum firing frequency, activation threshold current, or suppression threshold current of AP firing. One fascinating feature of the Aβ effect is its ability to alter the activation function in neurons, resulting in a multiplied input current. Specifically, Aβ1–42 can increase the input current by 30–70%, potentially indicating a boosted membrane resistance. However, this effect may also result in a slight hyperpolarization of the potential, requiring a slightly higher stimulation threshold to reach the generation threshold.

**FIGURE 6:**
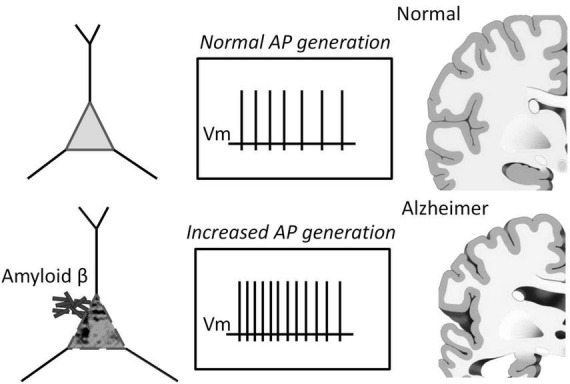
Schematic representation of the pathology of Alzheimer’s disease depicting the role of increased spiking of neurons in the presence of Aβ1–42.

We found that Aβ1–42 enhances impulse generation and lowers the firing threshold that can induce neuronal death at the long term influence, Figure 6. Our experiments confirm the effect of Aβ on reducing the rheobase of neuronal activation and on the increase in the frequency of firing under moderate stimulation. However, the precise mechanisms of these effects are still unknown. These secondary effects can influence and strengthen the recorded generation, and to describe the Aβ1–42 effect accurately, it is important to establish a common understanding of the terminology used. Our research suggests that the Aβ action may be equivalent to the amplification of the input current by 20–70%, effectively enhancing the neuronal response to input stimuli without significantly changing the shape and parameters of the activation function. However, in one-third of the neurons tested, we observed a different type of reaction to Aβ1–42, which led to a slight increase in firing frequency (5–15%), regardless of the stimulation intensity. This increase in activity did not involve any shift in the threshold current of the generation or changes in the AP suppression current. Thus, we have identified two distinct mechanisms of neuronal reaction to Aβ1–42: a slight gain in firing frequency or an increase in sensitivity to the input stimulus.

The study of temporal properties of the Aβ1–42 effect has shown that it develops in a few tens of seconds with a characteristic saturation time of nearly one minute. The latter gives evidence against the hypotheses of direct Aβ action as a ligand of voltage-depended channels but permits probable action through indirect signaling pathways, similar to metabotropic effects. It is also possible that Aβ may penetrate cells and adhere to the membranes of internal compartments, such as mitochondria or the endoplasmic reticulum. If this were to occur, it may still enhance the firing capacity of neurons for a few minutes. This enhancement would be marked by a prolonged generation of action potentials (APs) in response to a rectangular current pulse stimulation, with an improvement in the mechanism of rhythmogenesis in neurons that has not yet been fully established. Alternatively, it is plausible that the intracellular effects of penetrated Aβ may be compensatory and act as a programmable response of neurons through unidentified amyloid sensors.

The activation function can be a good tool for determining the pathways of influence of different substances. To conduct a comparative analysis of the activation functions of action potentials (APs) for amyloid and other substances, we can examine the direction and character of changes in the APs sequence. The absence of changes in the accommodation effect is a compelling argument that amyloid does not significantly alter sodium or calcium channels, or intracellular mechanisms of calcium signaling involved in the generation of APs. Impairment in sodium conductivity does not indicate a reduction in the amplitude of APs. Our previous research has demonstrated that additional calcium entry can significantly alter the slope of the APs sequence. In contrast, the action of Aβ1–42 is similar to that of the agonist of M1 muscarinic receptors McN-A-343 (Figure 5C). It is markedly different from the action of the voltage-gated sodium channel blocker quinidine, which reduces the frequency of the APs sequence by strengthening the accommodation, or from caffeine, which influences intracellular calcium signaling and leads to prolonged APs firing with low accommodation. Therefore, we can suppose that Aβ1–42 primarily affects impulse spiking through M-type (KCNQ) channels, rather than sodium or calcium channels.

The effect of Aβ1–42 is partially reversed at its high concentrations. In our experimental conditions, washing with the control solution after 10 min of Aβ application returned the APs activation function partially toward the control sequence by 20–50%. This backward effect can be considered a weak reduction of APs firing frequency at medium and high stimulus intensities. At the same time, under the condition of Aβ1–42 withdrawal, we observed impairment in the vitality of neurons with a decrease in the threshold of inhibition of generation and a general decrease in the rhythmicity of the pulse. Arrhythmia of AP generation can be a marker of irreversible degradation of neurons’ impulse activity and is always observed before the loss of the neuronal ability to fire, according to our previous experiments (Iavors’kyi and Luk”ianets’, 2013). The study suggests that the high concentrations of Aβ1-42 can partially reverse its effect on neurons. However, the withdrawal of Aβ1–42 leads to a decrease in the vitality of neurons, including a decrease in the threshold of inhibition of generation and a general decrease in the rhythmicity of the pulse. The arrhythmia of AP generation can be a marker of irreversible degradation of neurons’ impulse activity and is always observed before the loss of the neuronal ability to fire.

The present study has demonstrated that applying Aβ1–42 to isolated neurons leads to an increase in the frequency of AP generation and a shift in the threshold for AP generation. These findings agree with previous studies showing similar effects of Aβ1–42 on neuronal function (Jhamandas et al., 2011; George et al., 2021). It has been proposed that the interaction of Aβ1-42 with neuronal membrane receptors, such as N-methyl-D-aspartate (NMDA) and alpha-7 nicotinic acetylcholine receptors, may be responsible for the observed effects (Snyder et al., 2005; Dziewczapolski et al., 2009). This hypothesis is supported by the fact that these receptors are known to be involved in regulating AP generation and synaptic plasticity. Interestingly, the present study also found that the effects of Aβ1–42 were partly reversible upon washing, indicating that the observed changes in neuronal function were not due to irreversible structural damage. This finding is consistent with previous studies that have also reported reversible effects of Aβ1–42 on neuronal function (Shankar et al., 2007; Klyubin et al., 2008; Nimmrich et al., 2008; Ferreira et al., 2015).

In conclusion, our results provide further evidence for the involvement of Aβ in the modulation of neuronal function, particularly in the regulation of AP generation. These findings have implications for our understanding of the pathophysiology of Alzheimer’s disease and may provide insights into potential therapeutic targets for treating this devastating disorder.

## Data availability statement

The raw data supporting the conclusions of this article will be made available by the authors, without undue reservation.

## Ethics statement

This animal study was reviewed and approved by the National Academy of Sciences of Ukraine guidelines, Bogomoletz Institute of Physiology Committee for the care and use of laboratory animals.

## Author contributions

VY conducted the literature review and the first draft of manuscript writing, fulfilled electrophysiological experiments and analyses. EL fulfilled conceptualization of studies, created the study design, and contributed to manuscript revisions and translation. NR prepared solutions and tested amyloid. All authors contributed to the article and approved the submitted version.
